# The Relationship between Alcohol Outlets, HIV Risk Behavior, and HSV-2 Infection among South African Young Women: A Cross-Sectional Study

**DOI:** 10.1371/journal.pone.0125510

**Published:** 2015-05-08

**Authors:** Molly Rosenberg, Audrey Pettifor, Annelies Van Rie, Harsha Thirumurthy, Michael Emch, William C. Miller, F. Xavier Gómez-Olivé, Rhian Twine, James P. Hughes, Oliver Laeyendecker, Amanda Selin, Kathleen Kahn

**Affiliations:** 1 Center for Population and Development Studies, Harvard University, Cambridge, Massachusetts, United States of America; 2 Department of Epidemiology, University of North Carolina-Chapel Hill, Chapel Hill, North Carolina, United States of America; 3 Carolina Population Center, University of North Carolina-Chapel Hill, Chapel Hill, North Carolina, United States of America; 4 Department of Health Policy and Management, University of North Carolina-Chapel Hill, Chapel Hill, North Carolina, United States of America; 5 Department of Geography, University of North Carolina-Chapel Hill, Chapel Hill, North Carolina, United States of America; 6 Division of Infectious Diseases, Department of Medicine, School of Medicine, University of North Carolina-Chapel Hill, Chapel Hill, North Carolina, United States of America; 7 MRC/Wits Rural Public Health and Health Transitions Research Unit (Agincourt), School of Public Health, Faculty of Health Sciences, University of the Witwatersrand, Johannesburg, South Africa; 8 Department of Biostatistics, University of Washington, Seattle, Washington, United States of America; 9 Laboratory of Immunoregulation, NIAID, NIH, Baltimore, Maryland, United States of America; 10 Department of Medicine, Johns Hopkins University, Baltimore, Maryland, United States of America; 11 Centre for Global Health Research, Umeå University, Umeå, Sweden; 12 INDEPTH Network, Accra, Ghana; University of Washington, UNITED STATES

## Abstract

**Background:**

Alcohol consumption has a disinhibiting effect that may make sexual risk behaviors and disease transmission more likely. The characteristics of alcohol-serving outlets (e.g. music, dim lights, lack of condoms) may further encourage risky sexual activity. We hypothesize that frequenting alcohol outlets will be associated with HIV risk.

**Methods:**

In a sample of 2,533 school-attending young women in rural South Africa, we performed a cross-sectional analysis to examine the association between frequency of alcohol outlet visits in the last six months and four outcomes related to HIV risk: number of sex partners in the last three months, unprotected sex acts in the last three months, transactional sex with most recent partner, and HSV-2 infection. We also tested for interaction by alcohol consumption.

**Results:**

Visiting alcohol outlets was associated with having more sex partners [adjusted odds ratio (aOR), one versus zero partners (95% confidence interval (CI)): 1.51 (1.21, 1.88)], more unprotected sex acts [aOR, one versus zero acts (95% CI): 2.28 (1.52, 3.42)], higher levels of transactional sex [aOR (95% CI): 1.63 (1.03, 2.59)], and HSV-2 infection [aOR (95% CI): 1.30 (0.88, 1.91)]. In combination with exposure to alcohol consumption, visits to alcohol outlets were more strongly associated with all four outcomes than with either risk factor alone. Statistical evidence of interaction between alcohol outlet visits and alcohol consumption was observed for all outcomes except transactional sex.

**Conclusions:**

Frequenting alcohol outlets was associated with increased sexual risk in rural South African young women, especially when they consumed alcohol. Sexual health interventions targeted at alcohol outlets may effectively reach adolescents at high risk for sexually transmitted infections like HIV and HSV-2.

**Trial Registration:**

HIV Prevention Trials Network HPTN 068

## Introduction

Frequenting alcohol outlets (establishments where alcohol is sold and consumed) may influence sexual risk. Alcohol use and abuse is associated with increased sexual risk throughout the world,[1–5] regionally among populations in sub-Saharan Africa [6–8], and specifically in South Africa.[9,10] Additionally, characteristics of the outlets themselves (e.g. music, dim lights, lack of condoms) [11] and the network of people who typically frequent outlets (e.g. older men willing to exchange money or gifts for sex) [8,12] may create favorable environments for risky sexual activity. Relatedly, those inclined to risky sexual activity may visit alcohol outlets with the intention of engaging in risky behaviors.

Although the sexual risk profiles of individuals recruited at alcohol outlets have been characterized as risky,[8,13–17] the relative difference in sexual risk between those with and without alcohol outlet exposure is less clear. Adults who patronize alcohol outlets tend to engage in riskier sexual behaviors than those who do not.[18–21] However, this association has not been studied in adolescent-specific or female-specific populations.

Young women in South Africa have moderately high alcohol consumption [22,23] and are at exceptionally high risk for HIV infection.[24,25] Identifying novel risk factors and intervention targets for sexual risk will be critical to reduce the burden of HIV in this vulnerable population. Alcohol outlets are appealing as avenues for sexual risk reduction because adolescent access can be modified through regulation and parental education, and because they can serve as locations in which to deliver prevention interventions.[26,27] In this paper, we aim to examine the association between alcohol outlets and sexual risk in a population-based sample of rural South African young women.

## Methods

### Population

To explore the association between alcohol outlet visits and sexual risk, we conducted a cross-sectional analysis using baseline data from the HPTN 068 study.[28] HPTN 068 is a Phase III randomized trial in the rural Agincourt sub-district in the Mpumalanga province of South Africa where the Medical Research Council/Wits University Rural Public Health and Health Transitions Research Unit (Agincourt) has run a health and socio-demographic surveillance system since 1992.[29] The trial has the aim to determine whether cash transfers conditional on school attendance reduce HIV risk in young women. Between 2011–2012, a total of 2533 female adolescents, aged 13 to 20 years, currently enrolled in school, and not currently pregnant or married were enrolled in the study. Written informed consent from a primary caregiver and written informed consent/assent from each young woman were obtained prior to participation. Ethical approvals for the parent study and secondary analysis were provided by the Office of Human Research Ethics at the University of North Carolina-Chapel Hill (#10–1868; #13–2013). Further ethical approval for the parent study was provided by the University of the Witwatersrand’s Committee for Research on Human Subjects and the Mpumalanga Province Health Research and Ethics Committee.

### Variables

All variables were collected in HPTN 068 baseline biological testing and surveys. To minimize the bias that may arise from providing sensitive information to an interviewer, an ACASI (audio computer-assisted interviewing) component was incorporated into the survey. This component allowed participants to privately read and listen to audiotaped questions and log their responses in a computer, without directly interacting with an interviewer.

The exposure, *alcohol outlet visits*, was a count variable in response to the question: “How many times in the past six months have you been to a tavern/shebeen?” We examined the distribution and removed observations with implausibly high responses (n = 1) and responses with repeated single digits (i.e. 11, 22, 33) that were likely a result of unintentional double-striking in ACASI (n = 26). We categorized the exposure with dichotomous cutpoints (0 visits versus ≥1 visits) and categorical cutpoints to separate those with no exposure (0 visits), low exposure (1–5 visits—on average, fewer than one visit per month), and high exposure (≥6 visits—on average, one or more visits per month).

We examined four sexual risk outcomes associated with elevated HIV risk.[24,30] *Sex partners* was the reported number of sex partners in the last three months. *Unprotected sex acts* was constructed by subtracting the total number of condom-protected vaginal sex acts from the total number of vaginal sex acts over the last three months. For both count variables, those with no prior sexual activity received a zero value. We recoded two sex partner responses likely due to double striking based on corroborating information in the reported lifetime sex partner number. We recoded seven sex act responses likely due to double striking based on corroborating information in the reported number of protected sex acts. For all analyses, we categorized sex partners and unprotected sex acts each into three categories: zero, one, and greater than one, as there were few responses greater than two for either variable. *Transactional sex* was a dichotomous variable constructed from responses to questions regarding whether the participant had received money or gifts from her most recent sex partner and whether she felt obligated to have sex in return. Testing for *prevalent herpes simplex virus 2 (HSV-2) infection* was performed at baseline using Kalon HSV-2 gG2 ELISA (Kalon Biological, Ltd., Surrey, United Kingdom).[31] HIV status, though ascertained for each young woman at baseline, was not analyzed in this study due to the small number of prevalent infections and the likelihood that at least some of the cases were a result of perinatal, not sexual, transmission.

We also explored the influence of several key covariates. Specifically, we examined *age*, in years, at baseline; *education*, the grade in which the young woman was enrolled at baseline; *household size*, the total number of people living in the same dwelling as the participant; *primary caregiver relationship*, the relationship of the young woman to her primary caregiver: daughter, sibling, niece, grandchild, other; and *household socio-economic status (SES)*, a log-transformed measure of monthly household expenditures, per capita. We also calculated a dichotomous variable, *age for grade*, flagging young women who were older than they should be had they progressed linearly through school (e.g. above age 14 in grade 8). Finally, *alcohol consumption frequency* was defined categorically in response to the question: “How often do you drink alcohol?” with six responses ranging from “Never” to “More than once per week.” The variation of the distribution of each covariate across different levels of alcohol outlet exposure was assessed using chi-square tests for categorical covariates and ANOVA tests for continuous covariates.

### Statistical Analysis

We used logistic regression models to estimate the association between alcohol outlet visits and the dichotomous transactional sex and HSV-2 outcomes. We used multinomial logistic regression models to estimate the assocation between alcohol outlet visits and the categorized sex partner and unprotected sex act outcomes. To assess whether the observed results were driven by the lack of sexual risk outcomes among those not yet sexually active, we also ran each model in a restricted sample of those who had experienced sexual debut.

To control for potential confounding, we identified a minimally sufficient adjustment set from a directed acyclic graph. We then assessed the functional form for each covariate with each outcome separately and coded them as suggested by likelihood ratio tests. To test for interaction by alcohol consumption, we dichotomized both the alcohol outlet visits and alcohol consumption variables into “some”versus “no”exposure levels. We then included an interaction term between visits and consumption in each model. We used ordinal logistic regression models for the categorical outcomes to maximize statistical power and because the unstratified results suggested an ordered relationship. We compared the effect estimates among those with each risk factor alone to the effect estimate among those with both risk factors. We assessed the statistical significance of the interaction term in each model with likelihood ratio tests. Because interaction tests are typically underpowered, we considered p-values under 0.2 to be statistically significant. All analyses were performed in SAS statistical software, v9.1.2 (Cary, NC).

## Results

**Fig 1:**
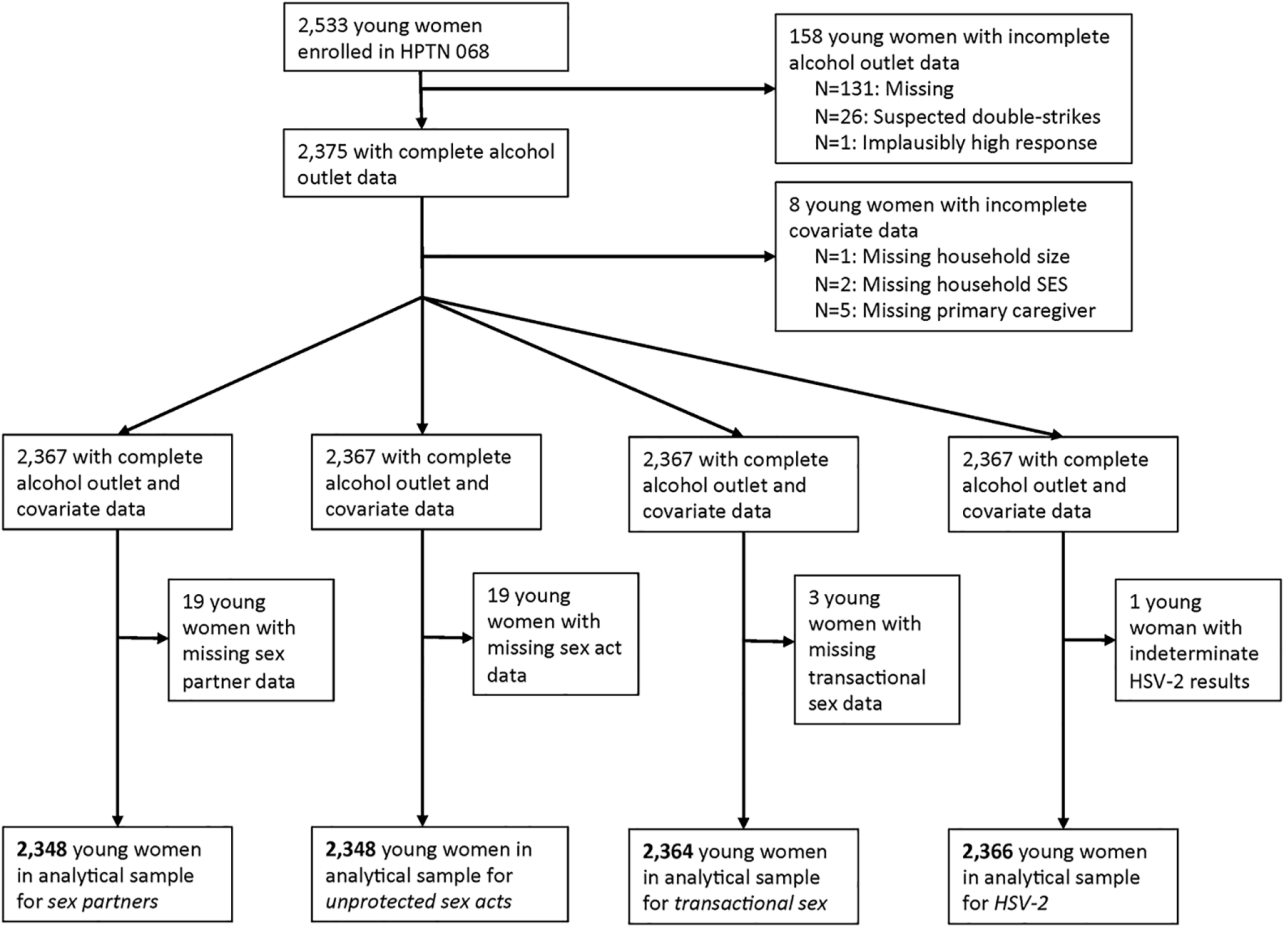
Flowchart of study sample construction for each of four sexual risk outcomes.

Overall, 2,533 young women were enrolled in HPTN 068 and completed baseline procedures (Fig 1). Only young women with complete exposure, outcome, and covariate data were included in the analyses. After removing observations with missing data for sex partners, unprotected sex acts, transactional sex, and HSV-2, respectively, a total of 2,348, 2,348, 2,364, and 2,366 young women remained in the analytical samples.

Over half (57%) of the young women reported no alcohol outlet exposure in the last six months, over one-third (38%) reported low levels of exposure, and 5% reported high levels of exposure (Table 1). The average age of participants (15.5 years), household size (6.2 people), and household SES (natural log of per capita expenditures: 5.2) did not vary significantly by level of alcohol outlet exposure. Most young women (74%) reported a mother or father as primary caregiver; these relationships were similar across levels of exposure. Current grade enrollment was significantly different across exposure frequencies. Those with higher exposure levels were more likely to be enrolled in lower grades (χ^2^ p-value: 0.003) and more likely to be older than the expected age for their grade level (χ^2^ p-value: 0.004).

**Table 1:** Demographic profile and sexual risk outcomes of 2375 rural, South African female adolescents,^a^ by frequency of alcohol outlet visits in the last six months, 2011–2012.

	Total(n = 2375)	No visits(n = 1354)	1–5 visits(n = 897)	≥6 visits(n = 124)	p-value
*Continuous variables*	*Mean (SD)*	*Mean (SD)*	*Mean (SD)*	*Mean (SD)*	*ANOVA Test*
**Age**	15.5 (1.7)	15.5 (1.6)	15.6 (1.7)	15.5 (1.8)	0.8
**Household size**	6.2 (2.6)	6.2 (2.7)	6.1 (2.5)	6.0 (2.7)	0.6
**Household SES** ^b^	5.2 (0.8)	5.2 (0.8)	5.2 (0.7)	5.2 (0.9)	0.8
*Categorical variables*	*N (%)*	*N (%)*	*N (%)*	*N (%)*	χ^2^ *Test*
**Education**					
Grade 8	603 (25.4)	331 (24.5)	233 (26.0)	39 (31.5)	0.003
Grade 9	632 (26.6)	334 (24.7)	253 (28.2)	45 (36.3)	
Grade 10	655 (27.6)	393 (29.0)	236 (26.3)	26 (21.0)	
Grade 11	485 (20.4)	296 (21.9)	175 (19.5)	14 (11.3)	
**Age for grade**					
Expected age or younger	1681 (70.8)	995 (73.5)	604 (67.3)	82 (66.1)	0.004
Older than expected age	694 (29.2)	359 (26.5)	293 (32.7)	42 (33.9)	
**Primary caregiver**					
Mother/Father	1756 (74.1)	985 (72.7)	680 (75.8)	91 (73.4)	0.8
Grandparent	331 (14.0)	199 (14.7)	116 (12.9)	16 (12.9)	
Sibling	160 (6.8)	93 (6.9)	56 (6.2)	11 (8.9)	
Aunt/Uncle	97 (4.1)	57 (4.2)	34 (3.8)	6 (4.8)	
Other	26 (1.1)	16 (1.2)	10 (1.1)	0 (0)	
**Alcohol consumption frequency**					
Never	2158 (91.0)	1273 (94.2)	786 (87.7)	99 (79.8)	<0.0001
< once a month	86 (3.6)	36 (2.7)	44 (4.9)	6 (4.8)	
Once a month	75 (3.2)	25 (1.9)	39 (4.4)	11 (8.9)	
2–3 times a month	20 (0.8)	9 (0.7)	8 (0.9)	3 (2.4)	
Once a week	19 (0.8)	5 (0.4)	13 (1.5)	1 (0.8)	
> once a week	15 (0.6)	4 (0.3)	6 (0.7)	4 (3.2)	
**Sexual debut**					
No	1729 (73.0)	1013 (75.1)	630 (70.5)	86 (69.4)	0.03
Yes	638 (27.0)	336 (24.9)	264 (29.5)	38 (30.7)	
**Number of sex partners** ^c^					
0	1794 (76.0)	1061 (79.0)	643 (72.0)	90 (72.6)	0.001
1	511 (21.7)	260 (19.4)	220 (24.6)	31 (25.0)	
2+	55 (2.3)	22 (1.6)	30 (3.4)	3 (2.4)	
**Number of unprotected sex acts** ^c^					
0	2176 (92.3)	1265 (94.4)	799 (89.5)	112 (90.3)	0.0002
1	78 (3.3)	36 (2.7)	39 (4.4)	3 (2.4)	
2+	103 (4.4)	39 (2.9)	55 (6.2)	9 (7.3)	
**Any transactional sex** ^d^					
No	2296 (96.7)	1319 (97.5)	859 (95.8)	118 (95.2)	0.05
Yes	78 (3.3)	34 (2.5)	38 (4.2)	6 (4.8)	
**HSV-2 infection**					
No	2259 (95.2)	1296 (95.7)	846 (94.4)	117 (94.4)	0.3
Yes	115 (4.8)	58 (4.3)	50 (5.6)	7 (5.7)	

^a^Sample restricted to those with non-missing alcohol outlet exposure data

^b^Household socio-economic status measured as natural log of per capita expenditures

^c^In the last three months

^d^With most recent partner

About one quarter of the young women reported sexual debut prior to baseline (27%). A similar proportion reported any sex partners (24%) and few reported any unprotected sex acts (8%) in the last three months. Just over 3% of young women reported transactional sex with their most recent partner and approximately 5% tested positive for HSV-2. The frequency of each outcome generally increased with increasing exposure. As expected, alcohol consumption frequency was higher with increasing alcohol outlet exposure (χ^2^ p-value: <0.0001); however, of the young women who reported visiting alcohol outlets, 87% reported no alcohol consumption.

Visiting alcohol outlets was positively associated with each sexual risk outcome (Table 2). Those reporting any alcohol outlet visits were more likely to report one versus zero [adjusted odds ratio (aOR) (95% confidence interval (CI)): 1.51 (1.21, 1.88)], and two or more versus zero sex partners [aOR (95%CI): 2.27 (1.29, 3.97)], compared to those with no visits. Similarly, those reporting any visits were more likely to report one versus zero [aOR (95%CI): 2.28 (1.52, 3.42)], and two or more versus zero unprotected sex acts [aOR (95%CI): 2.33 (1.53, 3.56)], compared to those with no visits. Alcohol outlet visits were also associated with increased transactional sex [aOR (95% CI): 1.63 (1.03, 2.59)] and prevalent HSV-2 infection [aOR (95%CI): 1.30 (0.88, 1.91)], though the CI around the HSV-2 estimate included the null. Generally, adjustment for confounding did not alter effect estimates appreciably from the unadjusted estimates. It is important to note, for rarer outcomes (transactional sex, HSV-2, and the highest category for sex partners and unprotected sex acts), the results were imprecise, with wide confidence intervals (confidence limit ratios above 2.0), and the confidence interval for the HSV-2 estimate included the null.

**Table 2:** The association between frequency of alcohol outlet visits in the last 6 months and behavioral and biologic sexual risk outcomes, among 2533 rural, South African female adolescents, 2011–2012.

		*Sex partners* ^*a*^ ^,^ ^*b*^		*Unprotected sex acts* ^*c*^ ^,^ ^*d*^		*Transactional sex* ^*e*^ ^,^ ^*f*^	*HSV-2 infection* ^*g*^ ^,^ ^*h*^
	Model	1 versus 0 partnersOR (95% CI)	2+ versus 0 partnersOR (95% CI)	1 versus 0 actsOR (95% CI)	2+ versus 0 actsOR (95% CI)	OR (95% CI)	OR (95% CI)
*Dichotomous exposure*	**Unadjusted**						
	0 visits	1	1	1	1	1	1
	≥1 visits	1.40 (1.15, 1.70)	2.17 (1.26, 3.75)	1.62 (1.03, 2.55)	1.69 (1.06, 2.70)	1.75 (1.11, 2.75)	1.32 (0.91, 1.92)
	**Adjusted**						
	0 visits	1	1	1	1	1	1
	≥1 visits	1.51 (1.21, 1.88)	2.27 (1.29, 3.97)	2.28 (1.52, 3.42)	2.33 (1.53, 3.56)	1.63 (1.03, 2.59)	1.30 (0.88, 1.91)
*Categorical exposure*	**Unadjusted**						
	0 visits	1	1	1	1	1	1
	1–5 visits	1.40 (1.14, 1.71)	2.25 (1.29, 3.93)	1.72 (1.08, 2.72)	2.23 (1.47, 3.40)	1.72 (1.07, 2.75)	1.32 (0.90, 1.95)
	≥6 visits	1.41 (0.91, 2.16)	1.61 (0.47, 5.47)	0.94 (0.29, 3.11)	2.61 (1.23, 5.52)	1.97 (0.81, 4.79)	1.34 (0.60, 3.00)
	**Adjusted**						
	0 visits	1	1	1	1	1	1
	1–5 visits	1.50 (1.19, 1.88)	2.37 (1.34, 4.20)	1.78 (1.10, 2.85)	2.27 (1.47, 3.50)	1.63 (1.01, 2.62)	1.29 (0.86, 1.93)
	≥6 visits	1.59 (0.97, 2.60)	1.56 (0.44, 5.54)	1.04 (0.30, 3.53)	2.88 (1.29, 6.39)	1.66 (0.66, 4.14)	1.37 (0.59, 3.20)

^a^Multinomial logistic regression model modeling the association between alcohol outlet visits and number of sex partners in the last three months, categorized as 0, 1, and 2+ partner

^b^Adjusted estimates are adjusted for age (coded with a quadratic term), current grade enrollment (coded with disjoint indicators for each grade), primary caregiver (coded dichotomously as parent versus non-parent), household size (coded linearly), and household SES (coded as deciles with a linear trend).

^c^Multinomial logistic regression model modeling the association between alcohol outlet visits and number of unprotected sex acts in the last three months, categorized as 0, 1, and 2+ acts

^d^Adjusted estimates are adjusted for age (coded linearly), current grade enrollment (coded with disjoint indicators for each grade), primary caregiver (coded dichotomously as parent versus non-parent), household size (coded linearly), and household SES (coded as deciles with a linear trend).

^e^Logistic regression model modeling the association between alcohol outlet visits and transactional sex with most recent sex partner

^f^Adjusted estimates are adjusted for age (coded with a quadratic term), current grade enrollment (coded linearly), primary caregiver (coded dichotomously as parent versus non-parent), household size (coded linearly), and household SES (coded as deciles with a linear trend).

^g^Logistic regression model modeling the association between alcohol outlet visits and prevalent HSV-2 infection

^h^Adjusted estimates are adjusted for age (coded linearly), current grade enrollment (coded linearly), primary caregiver (coded dichotomously as parent versus non-parent), household size (coded linearly), and household SES (coded with a quadratic term).

OR = odds ratio; CI = confidence interval

A dose-response relationship was not observed between number of alcohol outlet visits and any of the sexual risk outcomes. Using the more finely categorized alcohol outlet visit variable (0, 1–5, ≥6 visits over six months) tended to produce effect estimates of similar magnitude for both low (1–5 visits) and high (≥6 visits) exposure compared to no exposure. As a representative example, the odds ratios for transactional sex at both low [aOR (95%CI) = 1.63 (1.01, 2.62)] and high [aOR (95%CI) = 1.66 (0.66, 4.14)] exposure levels were qualitatively indistinguishable. Due to small numbers, results were imprecise for rarer outcomes and at the highest exposure level.

Visiting alcohol outlets was also positively associated with each sexual risk outcome in the restricted sample of 672 young women who had experienced sexual debut (Table 3). The results for each outcome were generally smaller but qualitatively similar in magnitude as those in the full sample. However, with the reduced sample size, estimates were less precise. For example, among the sexually active, those reporting any alcohol outlet visits were still more likely to report transactional sex, compared to those with no visits [aOR (95% CI): 1.43 (0.86, 2.36)].

**Table 3:** The association between frequency of alcohol outlet visits in the last 6 months and behavioral and biologic sexual risk outcomes, among 672 rural, South African female adolescents who experienced sexual debut prior to interview, 2011–2012

	*Sex partners* ^*a*^ ^,^ ^*b*^		*Unprotected sex acts* ^*c*^ ^,^ ^*d*^		*Transactional sex* ^*e*^ ^,^ ^*f*^	*HSV-2 infection* ^*g*^ ^,^ ^*h*^
Model	1 versus 0 partnersOR (95% CI)	2+ versus 0 partnersOR (95% CI)	1 versus 0 actsOR (95% CI)	2+ versus 0 actsOR (95% CI)	OR (95% CI)	OR (95% CI)
**Unadjusted**						
0 visits	1	1	1	1	1	1
≥1 visits	2.19 (1.29, 3.73)	3.20 (1.53, 6.72)	1.51 (0.93, 2.46)	2.19 (1.40, 3.41)	1.46 (0.89, 2.40)	1.28 (0.79, 2.06)
**Adjusted**						
0 visits	1	1	1	1	1	1
≥1 visits	2.08 (1.21, 3.58)	2.97 (1.39, 6.34)	1.47 (0.90, 2.41)	2.09 (1.33, 3.29)	1.43 (0.86, 2.36)	1.17 (0.71, 1.94)

^a^Multinomial logistic regression model modeling the association between alcohol outlet visits and number of sex partners in the last three months, categorized as 0, 1, and 2+ partners

^b^Adjusted estimates are adjusted for age (coded with a quadratic term), current grade enrollment (coded with disjoint indicators for each grade), primary caregiver (coded dichotomously as parent versus non-parent), household size (coded linearly), and household SES (coded as deciles with a linear trend).

^c^Multinomial logistic regression model modeling the association between alcohol outlet visits and number of unprotected sex acts in the last three months, categorized as 0, 1, and 2+ acts

^d^Adjusted estimates are adjusted for age (coded linearly), current grade enrollment (coded with disjoint indicators for each grade), primary caregiver (coded dichotomously as parent versus non-parent), household size (coded linearly), and household SES (coded as deciles with a linear trend).

^e^Logistic regression model modeling the association between alcohol outlet visits and transactional sex with most recent sex partner

^f^Adjusted estimates are adjusted for age (coded with a quadratic term), current grade enrollment (coded linearly), primary caregiver (coded dichotomously as parent versus non-parent), household size (coded linearly), and household SES (coded as deciles with a linear trend).

^g^Logistic regression model modeling the association between alcohol outlet visits and prevalent HSV-2 infection

^h^Adjusted estimates are adjusted for age (coded linearly), current grade enrollment (coded linearly), primary caregiver (coded dichotomously as parent versus non-parent), household size (coded linearly), and household SES (coded with a quadratic term).

OR = odds ratio; CI = confidence interval

Generally, alcohol outlet visits and alcohol consumption interacted to produce strong associations with the sexual risk outcomes (Table 4). The associations for each risk factor alone were predominantly positive, but small and not statistically significant. However, reporting both alcohol outlet visits and alcohol consumption, compared to neither visits nor consumption, was strongly associated with a higher number of sex partners [aOR (95%CI): 5.20 (3.54, 7.63)], a higher number of unprotected sex acts [aOR (95%CI): 4.39 (2.65, 7.28)], transactional sex [aOR (95%CI): 2.87 (1.38, 5.98)], and HSV-2 infection [aOR (95%CI): 2.44 (1.29, 4.59)]. Statistical evidence for improved model fit from the addition of the interaction term was observed in varying strengths for all outcomes except transactional sex.

**Table 4:** Interaction between alcohol outlet visits and alcohol consumption on sexual risk outcomes, among 2533 rural, South African female adolescents, 2011–2012.

		*Sex partners* ^*b*^ ^,^ ^*c*^		*Unprotected sex acts* ^*c*^ ^,^ ^*d*^		*Transactional sex* ^*c*^ ^,^ ^*e*^		*HSV-2 infection* ^*c*^ ^,^ ^*f*^	
AO visits	Alcohol consumption	aOR (95% CI)		aOR (95% CI)		aOR (95% CI)		aOR (95% CI)	
no	no	1		1		1		1	
yes	no	1.24 (0.99, 1.55)		1.72 (1.22, 2.50)		1.53 (0.91, 2.57)		1.10 (0.72, 1.70)	
no	yes	1.34 (0.77, 2.32)		1.36 (0.58, 3.22)		2.37 (0.88, 6.43)		0.98 (0.34, 2.87)	
yes	yes	5.20 (3.54, 7.63)		4.39 (2.65, 7.28)		2.87 (1.38, 5.98)		2.44 (1.29, 4.59)	
**LRT** ^a^ **statistics**		**Χ** ^**2**^	**p**	**Χ** ^**2**^	**p**	**Χ** ^**2**^	**p**	**Χ** ^**2**^	**p**
		3.1	0.0008	1.6	0.2	0.1	0.7	1.7	0.2

^a^LRT = Likelihood ratio test for whether the addition of the interaction term provides a significantly better fitting model compared to a model without the interaction term

^b^Ordinal logistic regression model estimating the association between each risk factor and number of sex partners categorized at 0, 1, and 2+ partners.

^c^Each adjusted estimate was adjusted for age, current grade enrollment, primary caregiver, household size, and household SES. All covariates were coded as noted above for each respective outcome.

^d^Ordinal logistic regression model estimating the association between each risk factor and number of unprotected sex acts categorized at 0, 1, and 2+ acts.

^e^Logistic regression model estimating the association between each risk factor and transactional sex with most recent partner.

^f^Logistic regression model estimating the association between each risk factor and prevalent HSV-2 infection.

AO = alcohol outlet; aOR = adjusted odds ratio; CI = confidence interval

## Discussion

We found that frequenting alcohol outlets was associated with increased sexual risk in rural South African young women, especially when they consumed alcohol. Young women who visited alcohol outlets reported more sex partners, more unprotected sex acts, and higher levels of transactional sex, and were more likely to have a prevalent HSV-2 infection, than young women who did not. The combination of exposure to both alcohol consumption and alcohol outlet visits was especially important—young women who reported both risk factors were much more likely to have experienced all four sexual risk outcomes, compared to those with neither risk factor.

This analysis provides important methodological improvements to and extends the generalizability of the current literature linking alcohol outlet visits to sexual risk.[18–21] To our knowledge, this is the first study to explore and confirm this association among adolescents, in particular adolescent women from sub-Saharan Africa, a vulnerable and high-risk population for HIV. Methodologically, this population was randomly sampled from within a health and socio-demographic surveillance site, yielding improved validity over studies using convenience sampling from within alcohol outlets.[20,21]

We also provide the first evidence that there is little or no dose-response relationship between alcohol outlet visits and sexual risk. All previous studies with an unexposed comparison group dichotomized the alcohol outlet exposure, potentially masking interesting variations in the relationship with sexual risk. However, we found that even those with alcohol outlet exposure levels fewer than six visits over six months had similarly elevated sexual risk outcomes as those with higher levels of alcohol outlet exposure. This lack of a dose-response runs counter to the dose-response relationship that has been observed in a population sampled from patrons of alcohol outlets.[14] These seemingly contradictory findings may be explained by differences in sample populations (a random sample of general population versus a sample defined by alcohol outlet visit history) and further highlight the need for more research on the relationship between alcohol outlets and sexual risk in a general population setting.

It is important to note that the level of exposure to alcohol outlets is generally low in this population: less than half of the entire sample reported any visits to alcohol outlets; among them, most reported fewer than six visits over a six month period. However, this is a population of minors who, legally, should not be visiting alcohol outlets, and we found that even adolescents at young ages were at risk for exposure. Even given this constraint, low levels of alcohol outlet exposure were robustly associated with indicators of sexual risk.

We also found that, with the exception of transactional sex, young women who visited alcohol outlets and consumed alcohol had stronger associations with all sexual risk outcomes than anticipated given the associations with each risk factor alone. Although the alcohol consumption and alcohol outlet visits, as recorded, may not necessarily have occurred at the same time, it is plausible to speculate that these doubly-exposed young women were consuming alcohol within the drinking establishments themselves. Consumption of alcohol leads to disinhibition, impaired decision-making, and feelings of reduced sexual control.[8,10,32–34] These disruptions to the normal constraints on risky activity lead to increased sexual risk.[35,36] In conjunction, characteristics typical of alcohol outlets (limited supervision, music, dim lights, unisex toilets, lack of condoms) may directly create favorable environments for risky sexual activity.[11] Sex partners are also often met in these places, particularly for young women, and these partners are often older men willing to exchange money or gifts for sex.[8,12] This combination of risk disinhibition from alcohol consumption with the risk opportunities presented within alcohol outlets may explain the heightened sexual risk observed among the doubly-exposed.

The observed associations could plausibly be driven by young women who had not yet experienced sexual debut. Our primary analysis included young women with and without prior sexual debut. Those who were not sexually active, and therefore precluded from experiencing any of the sexual risk outcomes, were less likely to visit alcohol outlets. However, the results from the sub-analysis restricted to those having experienced sexual debut suggest this is not the case. Visiting alcohol outlets appeared to have similar associations with each outcome among young women with sexual experience and among the full sample of young women.

It is also possible that we observe an association between alcohol outlet visits and sexual risk because young women inclined to sexual risk are also inclined to visit alcohol outlets, or because they visit alcohol outlets in order to meet like-minded partners or transactional sex partners. We attempted to minimize these possibilities by controlling for a set of covariates with hypothesized relationships to both exposure and outcome. However, the possibility remains that the observed association may be due to uncontrolled confounding.

The cross-sectional nature of the data does not allow us to assess the directionality of the observed association. In particular, we cannot say when the HSV-2 outcome occurred in relation to the alcohol outlet exposure. However, as this was a young cohort (mean age: 15.5), we expect that the dates of sexual debut and, therefore, earliest possible HSV-2 infection occurred relatively recently. Moreover, the information on sex partners, unprotected sex acts, and alcohol outlet exposure were collected with reference to the same three- to six-month time frame. The transactional sex outcome was restricted to refer to the most recent partner, so the timing was likely similar to the six-month exposure window as well. However, we acknowledge the possibility of a reverse causal relationship. Within the context of a risky partnership, the behavioral outcomes could lead to alcohol outlet visits (i.e. young women may visit taverns with their risky partners).

There were also several factors that could have led to data error. First, participants may have had difficulty remembering precise counts of activities, such as the exact number of sex partners, sex acts, or alcohol outlet visits that occurred over three and six months. Second, an unintended consequence of the ACASI data collection method is the potential for measurement error. The data suggested that some participants entered unintended responses. For this reason, we carefully examined the distributions of alcohol outlet visits, number of sex partners, and number of sex acts, and removed implausibly high responses and suspected double strikes. Third, as the exposure and behavioral outcomes were self-reported, it is plausible that willingness to report one risk behavior (alcohol outlet visits) was correlated with willingness to report another (sexual risk behavior). This concern is mitigated by the fact that an ACASI data collection method was employed to reduce the effects of social desirability bias, the associations remained after removing those who had not reported sexual debut (effectively removing those who were unwilling to report any risky behaviors), and similar associations, though generally smaller in magnitude and less precise, were observed with the objectively measured HSV-2 outcome.

In the context of the high HIV burden among South African female adolescents, identifying new risk factors and appropriate interventions for sexual risk is critical. This study suggests that young women who frequent alcohol outlets and consume alcohol have heightened sexual risk compared to those who do not. Consequently, alcohol outlets could be important places to reach high-risk adolescent women with sexual health interventions. Future studies that establish the directionality of the association will be able to inform whether HIV risk in adolescent women could be lowered by introducing interventions or policies to reduce their exposure to alcohol and alcohol outlets.[37,38]

## References

[1] Sales JM, Brown JL, Vissman AT, DiClemente RJ (2012) The association between alcohol use and sexual risk behaviors among African American women across three developmental periods: a review. Curr Drug Abuse Rev 5: 117–128. 22455508 10.2174/1874473711205020117 PMC3815711

[2] Li Q, Li X, Stanton B (2010) Alcohol use and sexual risk behaviors and outcomes in China: a literature review. AIDS Behav 14: 1227–1236. 10.1007/s10461-009-9648-5 19967440 PMC2943541

[3] Rehm J, Shield KD, Joharchi N, Shuper PA (2012) Alcohol consumption and the intention to engage in unprotected sex: systematic review and meta-analysis of experimental studies. Addiction 107: 51–59. 10.1111/j.1360-0443.2011.03621.x 22151318

[4] Fisher JC, Bang H, Kapiga SH (2007) The association between HIV infection and alcohol use: a systematic review and meta-analysis of African studies. Sex Transm Dis 34: 856–863. 18049422 10.1097/OLQ.0b013e318067b4fd

[5] Cook RL, Clark DB (2005) Is there an association between alcohol consumption and sexually transmitted diseases? A systematic review. Sexually Transmitted Diseases 32: 156–164. 15729152 10.1097/01.olq.0000151418.03899.97

[6] Kalichman SC, Simbayi LC, Kaufman M, Cain D, Jooste S (2007) Alcohol use and sexual risks for HIV/AIDS in sub-Saharan Africa: systematic review of empirical findings. Prev Sci 8: 141–151. 17265194 10.1007/s11121-006-0061-2

[7] Pithey A, Parry C (2009) Descriptive systematic review of sub-Saharan African studies on the association between alcohol use and HIV infection. SAHARA J (Journal of Social Aspects of HIV/AIDS Research Alliance) 6. 10.1080/17290376.2009.9724944 20485855

[8] Mataure P, McFarland W, Fritz K, Kim A, Woelk G, Ray S, et al (2002) Alcohol use and high-risk sexual behavior among adolescents and young adults in Harare, Zimbabwe. AIDS and Behavior 6: 211–219.

[9] Chersich MF, Rees HV (2008) Vulnerability of women in southern Africa to infection with HIV: biological determinants and priority health sector interventions. AIDS 22 Suppl 4: S27–40. 10.1097/01.aids.0000341775.94123.75 19033753

[10] Morojele NK, Kachieng’a MA, Nkoko MA, Moshia KM, Mokoko E, Parry CD, et al (2004) Perceived effects of alcohol use on sexual encounters among adults in South Africa. African Journal of Drug and Alcohol Studies 3: 1–20.

[11] Morojele NK, Kachieng’a MA, Mokoko E, Nkoko MA, Parry CD, Nkowane AM, et al (2006) Alcohol use and sexual behaviour among risky drinkers and bar and shebeen patrons in Gauteng province, South Africa. Social science & medicine 62: 217–227. 16054281 10.1016/j.socscimed.2005.05.031

[12] Watt MH, Aunon FM, Skinner D, Sikkema KJ, Kalichman SC, Pieterse D (2012) "Because he has bought for her, he wants to sleep with her": alcohol as a currency for sexual exchange in South African drinking venues. Soc Sci Med 74: 1005–1012. 10.1016/j.socscimed.2011.12.022 22326304 PMC3298605

[13] Sivaram S, Srikrishnan AK, Latkin C, Iriondo-Perez J, Go VF, Solomon S, et al (2008) Male alcohol use and unprotected sex with non-regular partners: evidence from wine shops in Chennai, India. Drug Alcohol Depend 94: 133–141. 10.1016/j.drugalcdep.2007.11.016 18187270 PMC2268872

[14] Parks KA, Hsieh YP, Collins RL, Levonyan-Radloff K, King LP (2009) Predictors of risky sexual behavior with new and regular partners in a sample of women bar drinkers. J Stud Alcohol Drugs 70: 197–205. 19261231 10.15288/jsad.2009.70.197 PMC2653606

[15] Kalichman SC, Simbayi LC, Vermaak R, Jooste S, Cain D (2008) HIV/AIDS risks among men and women who drink at informal alcohol serving establishments (Shebeens) in Cape Town, South Africa. Prev Sci 9: 55–62. 10.1007/s11121-008-0085-x 18264762

[16] Fritz KE, Woelk GB, Bassett MT, McFarland WC, Routh JA, Tobaiwa O, et al (2002) The association between alcohol use, sexual risk behavior, and HIV infection among men attending beerhalls in Harare, Zimbabwe. AIDS and Behavior 6: 221–228.

[17] Singh K, Sambisa W, Munyati S, Chandiwana B, Chingono A, Monash R, et al (2010) Targeting HIV interventions for adolescent girls and young women in Southern Africa: use of the PLACE methodology in Hwange District, Zimbabwe. AIDS Behav 14: 200–208. 10.1007/s10461-009-9572-8 19452272 PMC3966072

[18] Bassett MT, McFarland WC, Ray S, Mbizvo MT, Machekano R, van de Wijgert JH, et al (1996) Risk factors for HIV infection at enrollment in an urban male factory cohort in Harare, Zimbabwe. JAIDS Journal of Acquired Immune Deficiency Syndromes 13: 287–293. 10.1097/00042560-199611010-00012 8898675

[19] Lewis JJ, Garnett GP, Mhlanga S, Nyamukapa CA, Donnelly CA, Gregson S (2005) Beer halls as a focus for HIV prevention activities in rural Zimbabwe. Sex Transm Dis 32: 364–369. 15912083 10.1097/01.olq.0000154506.84492.61

[20] Cain D, Pare V, Kalichman SC, Harel O, Mthembu J, Carey MP, et al (2012) HIV risks associated with patronizing alcohol serving establishments in South African Townships, Cape Town. Prev Sci 13: 627–634. 10.1007/s11121-012-0290-5 22992872 PMC4540371

[21] Go VF, Solomon S, Srikrishnan AK, Sivaram S, Johnson SC, Sripaipan T, et al (2007) HIV rates and risk behaviors are low in the general population of men in Southern India but high in alcohol venues: results from 2 probability surveys. J Acquir Immune Defic Syndr 46: 491–497. 18077840 10.1097/qai.0b013e3181594c75 PMC2884173

[22] Meghdadpour S, Curtis S, Pettifor A, MacPhail C (2012) Factors associated with substance use among orphaned and non-orphaned youth in South Africa. Journal of Adolescence 35: 1329–1340. 10.1016/j.adolescence.2012.05.005 22704785

[23] Reddy S, James S, Sewpaul R, Koopman F, Funani N, Sifunda S, et al (2010) Umthente Uhlana Usamila—The South African Youth Risk Behavior Survey 2008. Cape Town.

[24] Pettifor AE, Rees HV, Kleinschmidt I, Steffenson AE, MacPhail C, Hlongwa-Madikizela L, et al (2005) Young people's sexual health in South Africa: HIV prevalence and sexual behaviors from a nationally representative household survey. AIDS 19: 1525–1534. 16135907 10.1097/01.aids.0000183129.16830.06

[25] UNAIDS. (2012) Global AIDS Response Progress Report, 2012: Republic of South Africa.

[26] Kalichman S (2009) Social and Structural HIV Prevention in Alcohol-Serving Establishments. Alcohol research & health: the journal of the National Institute on Alcohol Abuse and Alcoholism 33: 184–194. PMC3860505 23584060

[27] Weir SS, Pailman C, Mahlalela X, Coetzee N, Meidany F, Boerma JT (2003) From people to places: focusing AIDS prevention efforts where it matters most. AIDS 17: 895–903. 12660537 10.1097/01.aids.0000050809.06065.e0

[28] HIV Prevention Trials Network HPTN 068: Effects of Cash Transfer for the Prevention of HIV in Young South African Women.

[29] Kahn K, Collinson MA, Gomez-Olive FX, Mokoena O, Twine R, Mee P, et al (2012) Profile: Agincourt Health and Socio-demographic Surveillance System. Int J Epidemiol 41: 988–1001. 22933647 10.1093/ije/dys115 PMC3429877

[30] Freeman EE, Weiss HA, Glynn JR, Cross PL, Whitworth JA, Hayes RJ (2006) Herpes simplex virus 2 infection increases HIV acquisition in men and women: systematic review and meta-analysis of longitudinal studies. AIDS 20: 73–83. 16327322 10.1097/01.aids.0000198081.09337.a7

[31] Delany-Moretlwe S, Jentsch U, Weiss H, Moyes J, Ashley-Morrow R, Stevens W, et al (2010) Comparison of focus HerpesSelect and Kalon HSV-2 gG2 ELISA serological assays to detect herpes simplex virus type 2 antibodies in a South African population. Sexually transmitted infections 86: 46–50. 10.1136/sti.2009.036541 19837726 PMC2866038

[32] Pihl RO, Peterson JB, Lau MA (1993) A biosocial model of the alcohol-aggression relationship. Journal of Studies on Alcohol and Drugs: 128 8410954 10.15288/jsas.1993.s11.128

[33] Steele CM, Josephs RA (1990) Alcohol myopia: Its prized and dangerous effects. American Psychologist 45: 921 2221564 10.1037//0003-066x.45.8.921

[34] Graham K, West P, Wells S (2000) Evaluating theories of alcohol-related aggression using observations of young adults in bars. Addiction 95: 847–863. 10946435 10.1046/j.1360-0443.2000.9568473.x

[35] Zawacki T (2011) Effects of alcohol on women’s risky sexual decision making during social interactions in the laboratory. Psychology of Women Quarterly 35: 107–118.

[36] Davis KC (2010) The influence of alcohol expectancies and intoxication on men's aggressive unprotected sexual intentions. Experimental and clinical psychopharmacology 18: 418 10.1037/a0020510 20939645 PMC3000798

[37] Cook WK, Bond J, Greenfield TK (2014) Are alcohol policies associated with alcohol consumption in low-and middle-income countries? Addiction 109: 1081–1090. 10.1111/add.12571 24716508 PMC4107632

[38] Campbell CA, Hahn RA, Elder R, Brewer R, Chattopadhyay S, Fielding J, et al (2009) The effectiveness of limiting alcohol outlet density as a means of reducing excessive alcohol consumption and alcohol-related harms. Am J Prev Med 37: 556–569. 10.1016/j.amepre.2009.09.028 19944925

